# Association between trimethylamine N-oxide and prognosis of patients with myocardial infarction: a meta-analysis

**DOI:** 10.3389/fcvm.2024.1334730

**Published:** 2024-12-10

**Authors:** Xiuqing Li, Yubao Wang, Jie Xu, Kaili Luo, Tao Dong

**Affiliations:** ^1^ Department of Gastroenterology and Hepatology, The Third People’s Hospital of Zhenjiang, Zhenjiang, Jiangsu, China; ^2^ Department of Cardiology, The Affiliated Lianyungang Oriental Hospital of Kangda College of Nanjing Medical University, Lianyungang, China

**Keywords:** trimethylamine N-oxide, myocardial infarction, all-cause mortality, meta-analysis, major adverse cardiovascular events

## Abstract

**Background:**

Trimethylamine N-oxide (TMAO) has been widely explored and considered as a biomarker for adverse cardiovascular events. However, the relationships between TMAO adverse cardiovascular events are inconsistent in patients. Therefore, this meta-analysis aimed to estimate association between TMAO levels and the prognosis of patients with myocardial infarction (MI).

**Methods:**

We searched PubMed, EMBASE, the Cochrane Library, and Web of Science from inception to July 2, 2023, to retrieve all relevant clinical trials. Associations between TMAO levels, major adverse cardiovascular events (MACE), all-cause mortality, recurrent MI, stroke, etc., were systematically addressed. Outcomes included MACE, all-cause mortality, recurrent MI, rehospitalization caused by heart failure, stroke, revascularization, SYNTAX score, and multivessel disease. A fixed/random-effects model should be adopted to calculate the pooled estimates. Besides, funnel plot, Begg's test and Egger' test were used to test publication bias.

**Results:**

A total of nine studies were included in our meta-analysis. Our results indicated that higher TMAO levels were associated with greater risk of MACE (RR = 1.94; 95% CI = 1.39 to 2.73), all-cause mortality (RR = 1.56; 95% CI = 1.00 to 2.44), and MI (RR = 1.21; 95% CI = 1.01 to 1.45). No significant association was found in stroke, SYNTAX, and multivessel disease. Besides, our results reported that the association between TMAO levels and MACE after MI was not affected by the geographic localization.

**Conclusion:**

This study was the first meta-analysis that showed a significant positive association of TMAO levels with MACE, all-cause mortality, and recurrent MI in patients with MI.

**Systematic Review Registration:**

https://www.crd.york.ac.uk/prospero/display_record.php?RecordID=460400, PROSPERO (CRD42023460400).

## Introduction

The prevalence and mortality of cardiovascular disease (CVD) are increasing worldwide, especially acute myocardial infarction (AMI) caused by the rupture of coronary atherosclerotic plaques, which is the leading cause of mortality worldwide (1). In the last decade, CVD deaths have increased by 12.5 percent globally (2). In China, the number of people with cardiovascular disease has reached 330 million (3), and high rates of recurrent ischemic events was observed worldwild [including recurrent AMI, cardiovascular mortality, and stroke (4)]. Trial results have shown that approximately 50% of patients with ST-segment elevation myocardial infarction (STEMI) have multivessel coronary artery disease that may lead to poor prognosis at the time of primary percutaneous coronary intervention (PCI) (5, 6). Unfortunately, sufficient consideration has been given to reducing traditional risk factors such as hyperlipidemia, smoking, hypertension, and diabetes mellitus; novel pharmacotherapy treatments only reduced 30% of CVD-related adverse outcomes (7). Therefore, it mainly challenges predicting adverse cardiac events among AMI patients. Also, identifying novel pathogenic risk factors related to CVD after myocardial infarction (MI) has vital clinical significance for disease prevention and early stratification (8).

Emerging studies reported that metabolites of gut microbiota were significantly associated with CVD. Trimethylamine-N-oxide (TMAO) is an intestinal-derived bacterial metabolite, and some gut microbiota produces trimethylamine cleaving enzymes that convert dietary directly ingested or indirectly made choline, betaine, carnitines, and TMAO to trimethylamine, which enters the liver via the portal circulation and is oxidized by flavin monooxygenase to produce TMAO (9, 10). Trimethylamine is absorbed into the bloodstream and rapidly oxidized to TMAO by hepatic flavin monooxygenase3 (11). Serum TMAO concentration ≥8.74 µM is considered to be a predictor of metabolic syndrome (12). Several studies have shown that high TMAO levels accelerate the progression of atherosclerotic plaques and are also associated with unstable plaques, rupture (13), and long-term risk of cardiovascular events in patients with acute coronary syndrome (14). TMAO triggers oxidative stress by inhibiting the SIRT3-SOD2-mitochondrial reactive oxygen species signaling pathway, activating thromboxane NIPNLRP3-type inflammatory vesicles (15), increasing the expression of cell-surface CD36 receptors, exacerbating macrophage and cholesterol accumulation, releasing the inflammatory cytokines interleukins 1, *β*, and 18, and inhibiting the production of nitric oxide synthase and nitric oxide, which in turn induces inflammation and endothelial dysfunction (16), promotes atherosclerosis, and participates in the development of AMI. TMAO has been widely explored and considered a biomarker for adverse cardiovascular events. A series of research (17–19) found that elevated circulating levels of TMAO independently predicted the risk of major adverse cardiac events, including stroke, myocardial infarction, and death. Also, levels of choline, betaine, and carnitine have been shown to be associated with the development of cardiovascular disease and to predict the risk of major adverse cardiovascular events (MACE) (20). A previous meta-analysis (21) showed a positive dose-dependent relationship between TMAO levels and increased cardiovascular risk and mortality.

Some meta-analyses have explored the association between TMAO levels and cardiovascular risks (21–27). For example, Yao et al. (25) found a significant relationship between TMAO levels and MACE in coronary heart disease (CHD) patients. Li et al. (27) also reported that TMAO could predict the risk of all-cause mortality in patients with chronic kidney disease (CKD). However, no meta-analysis was performed on the association of TMAO and MACE and death events in MI patients. Increasingly, studies explored the associations between TMAO levels and the prognosis of patients with MI (28–31). For example, Li et al. (28) demonstrated that TMAO levels during follow-up could identify changes in MACE risk in patients with AMI. Besides, Zhou et al. (31) found that increased plasma concentration of TMAO is an independent predictor of all-cause mortality. However, other studies made inconsistent conclusions. Suzuki et al. (30) indicated that TMAO was not an independent predictor of all-cause mortality after AMI, while TMAO could independently predict recurrent MI. Given the above research, their associations in patients with MI were inconsistent. Therefore, we performed the first meta-analysis of published studies to assess the relationship between TMAO levels and the prognosis of patients with MI. Thereby, the comprehensive evidence provided by this meta-analysis is important for an in-depth understanding of the relationship between TMAO and MI prognosis as well as its clinical application.

## Methods

The present meta-analysis was performed and reported according to the Preferred Reporting Items for Systematic Reviews and Meta-analyses (PRISMA) (32).

### Search strategy

We searched PubMed, EMBASE, the Cochrane Library, and Web of Science from inception to July 2, 2023, to retrieve all relevant clinical trials. Search keywords included “Gastrointestinal Microbiome”, “trimethylamine N-oxide”, and “Myocardial Infarction”. Also, we recovered the reference lists of relevant reviews by manual screening.

### Selection criteria

The selection criteria in this meta-analysis were generated based on the PICOS principle as follows.

### Inclusion criteria

Population: Adult patients diagnosed with MI (age ≥ 18 years old).

Outcome: Quantifiable relationship between changes in TMAO levels and prognosis in MI patients, including all-cause mortality, recurrent MI, rehospitalization caused by heart failure, stroke, revascularization, and SYNTAX score.

Study design: Both prospective and retrospective trials are acceptable.

Exclusion criteria:

(a) ineligible study design, such as case reports, commentary, and conference abstracts.
(b) essential data were absent from studies although emailed authors to obtain them.
(c) repeatedly published studies.
(d) reviews, protocol, and animal trials.

### Screening and data extraction

The screening process is divided into three steps: removing duplicate reports, browsing through the titles and abstracts, and assessing the full text's suitability. Two independent reviewers and disagreements assessed each study were resolved by discussion with a third reviewer.

Independent researchers worked in pairs to extract data, and inconsistencies were resolved by discussion or by having a third reviewer. We pulled information including the name of the first author, year of publication, study location, participant' age, male proportion (%), follow-up time, TMAO level, adjustment model (yes/no), and outcomes, including MACE (a composite of all-cause death, recurrence of MI, rehospitalization caused by HF, ischemic stroke, and any revascularization), SYNTAX score [A rating system that standardizes the complexity of lesions based on the anatomical features of the diseased coronary artery helps clinicians establish a scoring system for the best revascularization approach in patients with complex coronary artery disease (33)], and multivessel disease.

### Study quality assessment

Two authors independently adopted the Newcastle-Ottawa Scale (NOS) (34) and Jadad Score (35) to assess the quality of included articles. For NOS, the score range of this scale is 0 to 9, and a higher score indicates better methodological quality. The NOS score < 7 is defined as low quality and a score ≥ 7 as high quality. For Jadad Score, the quality scale ranges from 0 to 5 points, with a score ≤ 2 indicating a low quality report, and a score of ≥3 indicating a high quality report. The discussion among all authors is to solve disagreements.

### Statistical analysis

This meta-analysis used Stata V.14.0 (StataCorp LP). The outcomes were shown as RR with their 95% CI. The Cochrane Q and *I*^2^ statistics test the heterogeneity among all studies. If the value of *I*^2^ < 25 is regarded as the absence of heterogeneity, 25 to 50 is possible heterogeneity, and ≥50 is significant heterogeneity. When there is the presence of heterogeneity, a random-effects model should be adopted to calculate the weighted pooled RR and 95% CI using DerSimonian-Laird method (36). In addition, this study conducted a subgroup analysis to explore the moderator factors. A sensitivity analysis was performed to test the robustness of the pooled results. Funnel plot, Begg's test and Egger' test is to test publication bias. If the funnel plot is asymmetric, then there is the presence of publication bias. Egger' test uses a linear regression approach to interpret the asymmetric funnel plots. The probability value below 0.05 with two-tail was regarded as statistical significance.

## Results

### Study selection

**Figure 1:**
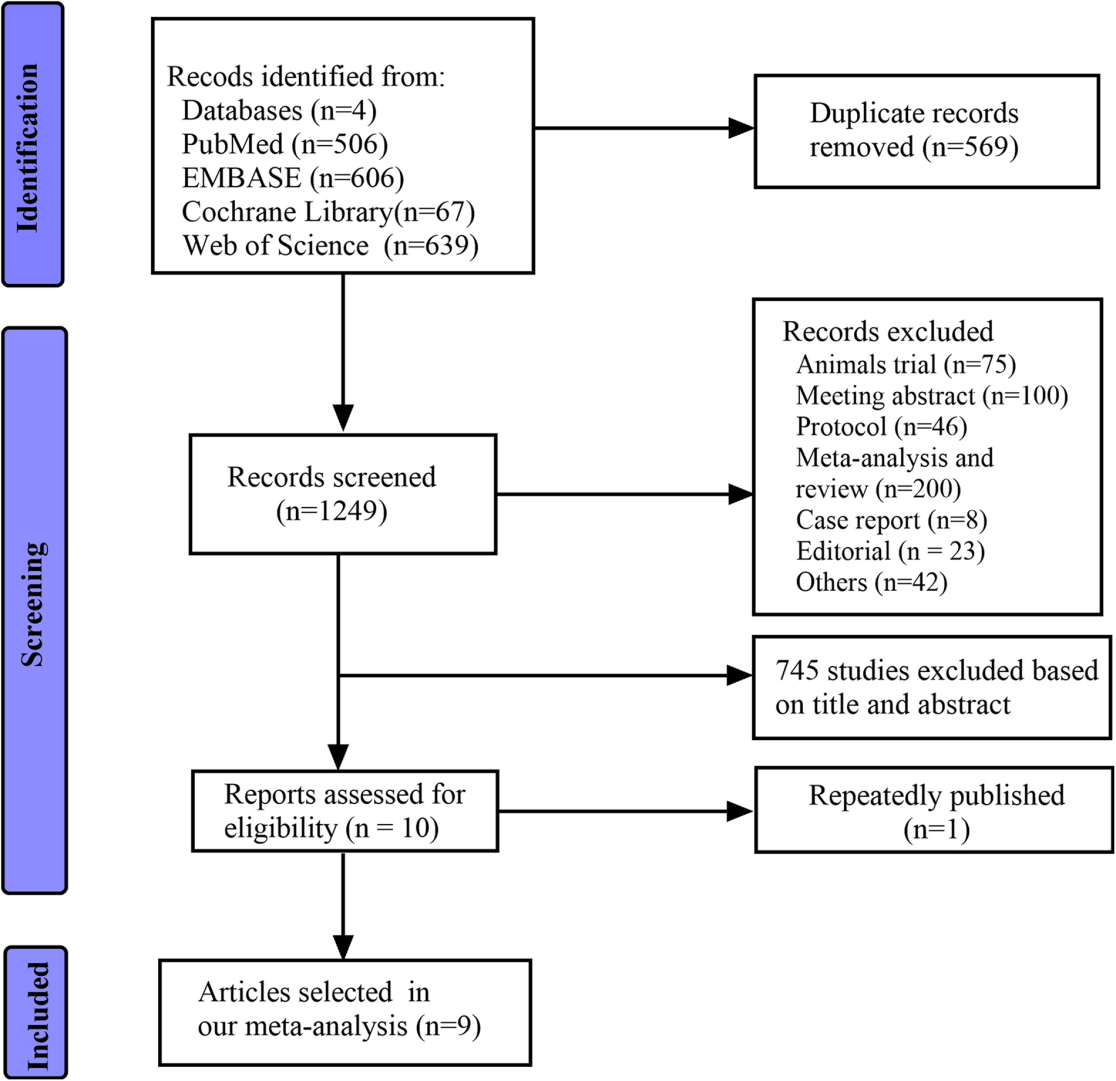
Flow diagram of the search process and study selection.

In sum, 1,818 articles were identified in electronic and manual searches. However, 569 articles were excluded for duplication, and another 1,239 articles were excluded due to study types (reviews, meeting abstracts, animal trials, case reports, and others) and irrelevance. Finally, ten articles were reviewed in full text based on inclusion. Two studies (28, 29) used the same cohort. Therefore, nine non-randomised studies (28, 30, 31, 37–42) were included in the current meta-analysis (Figure 1).

### Study characteristics

This meta-analysis included eight prospective studies and one retrospective study. Among the selected studies, five studies were conducted in China and 1 in USA, the UK, the Republic of Lithuania, and Poland, respectively. The sample sizes of participants ranged from 84 to 1,803. Besides, all participants' age was 60 and above, and the majority of participants were male with 70.2%. The follow-up time ranged from 12 to 42 months. The characteristics of eligible studies are listed in Table 1.

**Table 1:** Basic characteristics of all the included studies.

Study	Location	Study design	Sample size	Age (year)	Male (%)	Follow-up (month)	TMAO level	Adjustment model	Outcomes	NOS
Li et al. (28)	China	Prospective	509	61 (53.4, 60.6)	423 (83.1)	12	>12.7 μmol	Yes	MACE	7
Gencer et al. (38)	USA	Prospective	1,803	≥65	1,343 (74.4)	33	>7.22 μmol	Yes	MACE	8
Gąsecka et al. (37)	Poland	Prospective	84	64.4	42 (73.7)	42	478 ng/ml	Yes	MACE	8
Zhao et al. (39)	China	Retrospective	1,004	63.6 ± 11.3	700 (69.7)	12	>2.86 μmol	Yes	MACE	7
Sheng et al. (40)	China	Prospective	388	58.7 ± 12.1	270 (80.6)	>12	>2.46 μM	Yes	SYNTAX score and multivessel disease	8
Aldujeli et al. (41)	Republic of Lithuania	Prospective	200	65.5 (58, 76)	81 (40.5)	12	NR	Yes	new-onset atrial fibrillation	8
Zhou et al. (31)	China	Prospective	1,208	73 (64, 80)	828 (68.5)	22.4	>7.92 μmol	Yes	MACE	8
Suzuki et al. (30)	UK	Prospective	1,079	67 (57, 77)	777 (72.0)	24	>5.1 μmol	Yes	All-cause mortality and recurrent MI	7
Waleed et al. (42)	China	Prospective	108	60.1 ± 12.1	61 (69.3)	NR	NR	Yes	SYNTAX score and multivessel disease	9

NOS, Newcastle-Ottawa scale; TMAO, trimethylamine N-oxide; MACE, major adverse cardiovascular events.

### Study quality

We adopted the NOS to assess the quality of concerning studies. Therefore, all studies' scores were equal to or higher than seven, which was considered high quality. The results of the study quality are shown in Table 1.

### Association of TMAO with MACE, all-cause mortality, recurrent MI and stroke after MI

**Figure 2:**
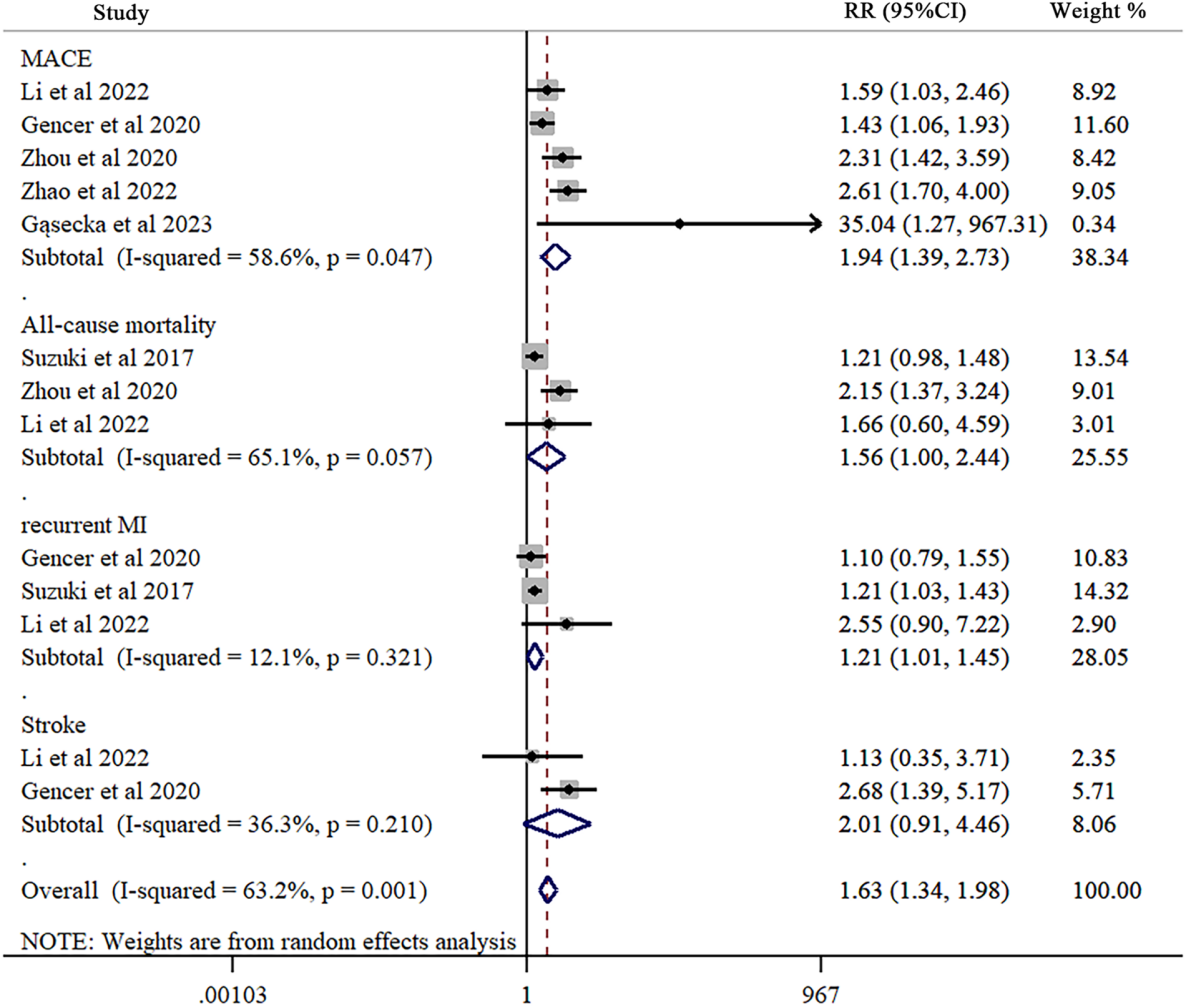
Forest plot of trimethylamine-N-oxide levels in major adverse cardiovascular events, all-cause mortality, recurrent myocardial infarction, and stroke after myocardial infarction. Each study is represented by a square and a horizontal line, which represents its relative risk and corresponding 95% CI, respectively. The area of the square is proportional to the weight of the study in the pooled analysis. The studies are sorted by weight in the plot and study design. The pooled random-effects estimate and its 95% CI are represented by a red dashed vertical line and diamond. The vertical black line at 1.0 indicates no effect of TMAO on major adverse cardiovascular events, all-cause mortality, recurrent myocardial infarction, and stroke after myocardial infarction.

Across all studies, five studies examine the association of TMAO with MACE after MI. Our results indicated that patients with higher TMAO levels had 1.94-fold increased MACE risk (RR = 1.94; 95% CI = 1.39 to 2.73; *I*^2^ = 58.6%, *P* < 0.047) (Figure 2). When the analysis for MACE was repeated, stratifying the studies according to the study design (prospective and retrospective), a significant association between TMAO levels and MACE was found in both two groups (prospective: 4 studies; RR = 1.76; 95% CI = 1.22 to 2.53; *I*^2^ = 51.5%, *P* = 0.103; retrospective: 1 study; RR = 2.61; 95% CI = 1.70 to 4.00) (Supplementary Figure S4A). Subsequently, sub-group analysis was performed according to the geographical location. The association between MACE and TMAO levels was also statistically significant across different countries (USA: 1 study; RR = 1.43; 95% CI = 1.06 to 1.93; China: 3 studies; RR = 2.12; 95% CI = 1.58 to 2.86; *I*^2^ = 26.4%, *P* = 0.257; Poland: 1 study; RR = 35.04; 95% CI = 1.27 to 967.45) (Supplementary Figure S4B).

Three examine the association between TMAO and all-cause mortality after MI. Significant relationship between them was found (RR = 1.56; 95% CI = 1.00 to 2.44; *I*^2^ = 65.1%, *P* = 0.057) (Figure 2).

Three explored the association between TMAO levels and recurrent MI after MI. TMAO and recurrent MI had a significant association after MI (RR = 1.21; 95% CI = 1.01 to 1.45; *I*^2^ = 12.1%, *P* = 0.321) (Figure 2).

Two studies examined the association between TMAO and stroke after MI. Our results reported no significant association (RR = 2.01; 95% CI = 0.91 to 4.46; *I*^2^ = 36.3%, *P* = 0.210) (Figure 2).

### Association of TMAO with SYNTAX score and multivessel disease after MI

**Figure 3:**
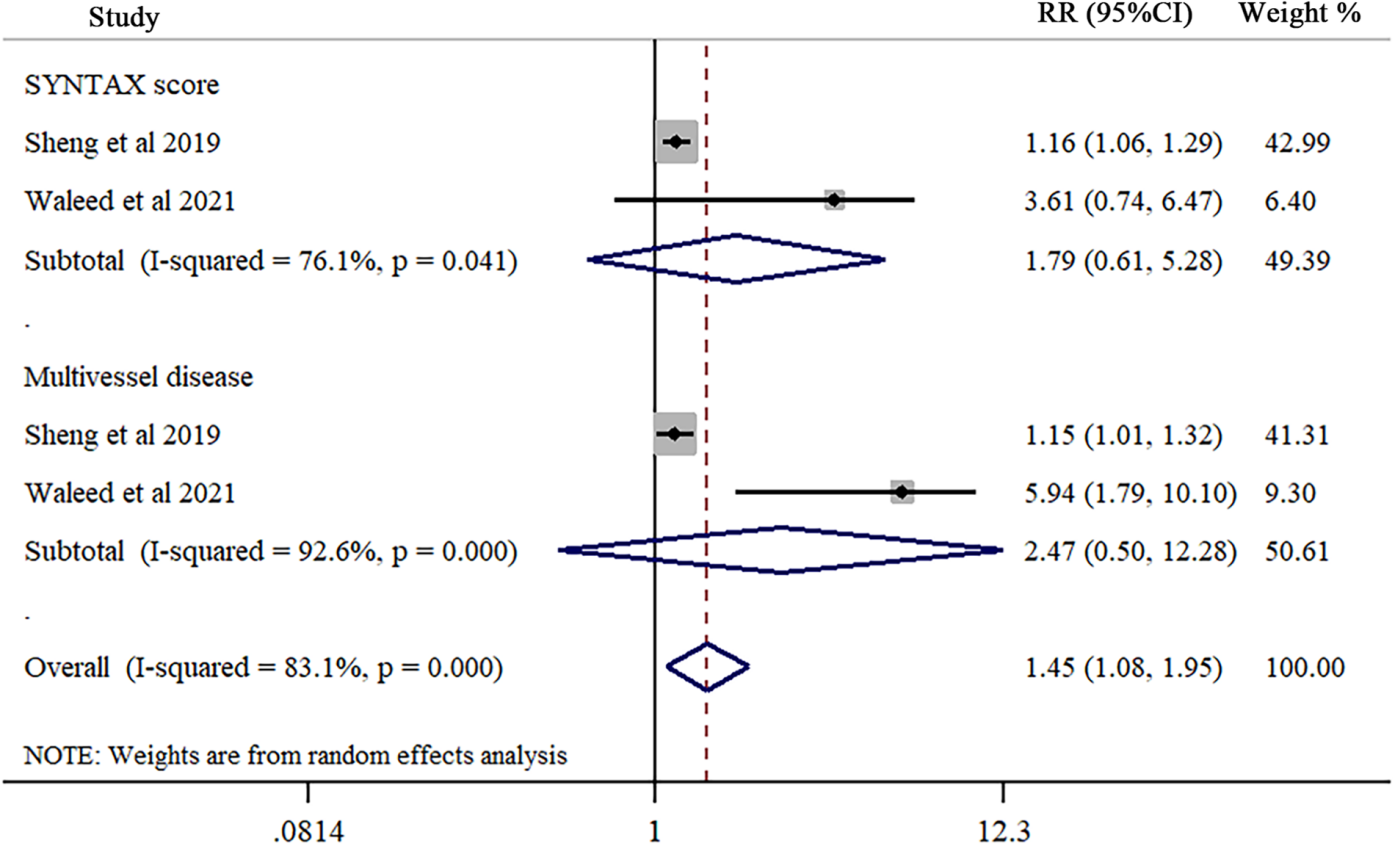
Forest plot of trimethylamine-N-oxide levels in SYNTAX score and multivessel disease after myocardial infarction. Each study is represented by a square and a horizontal line, which represents its relative risk and corresponding 95% CI, respectively. The area of the square is proportional to the weight of the study in the pooled analysis. The studies are sorted by weight in the plot and study design. The pooled random-effects estimate and its 95% CI are represented by a red dashed vertical line and diamond. The vertical black line at 1.0 indicates no effect of TMAO on SYNTAX score and multivessel disease after myocardial infarction.

Two studies explored the association of TMAO with SYNTAX score and multivessel disease, respectively. Our results reported that no significant association was found in SYNTAX (RR = 1.79; 95% CI = 0.61 to 5.28; *I*^2^ = 76.1%, *P* = 0.041) (Figure 3) and multivessel disease (RR = 2.47; 95% CI = 0.50 to 12.28; *I*^2^ = 92.6%, *P* < 0.001) (Figure 3).

Besides, one study examined the association of TMAO with recurrent heart failure (RR = 0.94; 95% CI = 0.16 to 5.51), new-onset atrial fibrillation (RR = 1.29; 95% CI = 1.00 to 1.66), cardiovascular death (RR = 2.25; 95% CI = 1.28 to 3.96), and revascularization (RR = 2.21; 95% CI = 1.28 to 3.80), respectively.

### Sensitivity analysis and publication bias

Results of sensitivity analysis indicated that all the pooled estimates were stable, which stated that our results were reliable (Supplementary Figures S1–S3). When repeating the meta-analysis, deleting one study at a time confirmed that the incidence of MACE, all-cause mortality, and recurrent MI was significantly higher in the high TMAO level group than in the low TMAO level group.

Furthermore, the funnel plot indicated no publication bias (Supplementary Figure S5). Also, both Begg test and Egger test did not show publication bias in MACE (Begg's test, *P* = 0.327; Egger's test, *P* = 0.135), all-cause mortality (Begg's test, *P* = 0.602; Egger's test, *P* = 0.536), and recurrent MI (Begg's test, *P* = 0.602; Egger's test, *P* = 0.547).

## Discussion

This study is the first meta-analysis to examine the relationship between TMAO levels and the prognosis of MI patients. The main finding is that high levels of TMAO are significantly associated with an increased risk of MACE including all-cause mortality, and recurrent MI, indicating that TMAO may be an effective biomarker for adverse cardiovascular events. There may be a potential synergistic effect between TMAO and inflammation on cardiovascular risk (29).

Formerly published meta-analyses have presented that high TMAO increased the risk of MACE or death events among patients with chronic diseases (25, 27, 43), such as heart failure, CHD, and CKD, consistent with our results. Besides, a previous meta-analysis reported that TMAO level could not significantly predict stroke (HR = 1.01; 95% CI = 0.84 to 1.22). Similar results (RR = 2.01; 95% CI = 0.91 to 4.46) were found in the present study. Therefore, these results must be considered carefully and further investigated because only two articles were included to examine the relationship between TMAO level and stroke in both studies. Furthermore, other studies (23) demonstrated that TMAO levels could predict other health conditions, including hypertension, diabetes mellitus (DM), cancer, and kidney function. Whether TMAO is, only a marker of cardiovascular disease or even a mediator remains unclear. However, TMAO may be involved in mechanisms and phases of the otosclerosis process and complications (44, 45). TMAO, a bioactive molecule that accelerates atherosclerotic plaque progression, is associated with unstable plaques, rupture (13), and the risk of long-term cardiovascular events in patients with acute coronary syndrome (14). An increasing number of studies suggest that TMAO may be a promising cardiovascular risk marker. Therefore, TMAO may partially explain the long-standing puzzle of residual cardiovascular risk.

Our results reported significant heterogeneity. This meta-analysis performed sensitivity and sub-group analysis to investigate the source of heterogeneity. First, the results of our study indicated that our results were stable. In other words, the correlations between TMAO level and MACE, all-cause mortality, and MI recurrence were significant and stable. Based on the study design, the included studies were classified into two groups: prospective and retrospective. The *I*^2^ for MACE was relatively low. At the same time, only one study was retrospective. Hence, the different study designs included in the study may be sources of variability explaining the relationship between TMAO and MACE in MI patients.

Furthermore, our results reported that the geographical location of the population in the study did not affect the relationship between TMAO levels and MACE after MI. Individual distribution of TMAO blood concentration might be affected by different consumption of TMAO-producing foods. Some studies indicated that TMAO is a circulating metabolic product produced by the gut microbiota, involving abundant nutritional precursors in Western diets (46–48). However, our findings should be taken cautiously due to the limited number of studies (one study in the USA and Poland, respectively). Besides, compared with retrospective studies, our subgroup analysis did not show any significant differences in MACE in prospective studies.

In the current study, only two studies in our meta-analysis examined the association of TMAO levels with SYNTAX score and multivessel, respectively. Besides, one study examined the association of TMAO with recurrent heart failure, new-onset atrial fibrillation, cardiovascular death, and revascularization, respectively. Therefore, these results need to be considered carefully and further investigated.

## Implications

Our results indicated that decreasing TMAO is a worthwhile intervention strategy to explore. Effective interventions to alter the gut microbiota composition and thus reduce TMAO production or increase TMAO clearance can effectively prevent the development of CVD. However, studies exploring the effects of diet, drugs, and lifestyle on flora in populations need better and more credible data. More in-depth studies are required to unravel the mechanisms of action of gut microbiota and their metabolites. The severity of CVD can be assessed by identifying individuals with elevated cardiovascular risk through the detection of TMAO levels in asymptomatic populations and by monitoring changes in TMAO levels, and the correlation between gut microbiota and TMAO can be analyzed. The correlation between gut microbiota and TMAO will be analyzed, and relevant bacteria will be selected for targeted therapy, opening up a new direction for preventing and treating CVD. TMAO is a small organic compound that is formed by the oxidation of trimethylamine in the host liver by flavin monooxygenases. In the body, foods rich in choline, lecithin, and L-carnitine, such as red meat, eggs, dairy products, and salted fish, are metabolized by the gut microbiota to produce the precursor trimethylamine (49). Koeth et al. (50) found that plasma levels of choline, betaine, and TMAO were associated with an increased risk of CVD, and the formation of TMAO was predictive, especially choline and L-carnitine. Given this, non-pharmacological strategies can regulate the gut microbiota to reduce TMAO levels, thereby reducing the risk of cardiovascular disease, including diets and food supplements rich in bioactive compounds or nutrients.

## Limitations

Current meta-analysis should pay attention to several limitations. Firstly, the number of studies included in our meta-analysis is relatively small. For example, only three studies examined the relationships between TMAO levels and all-cause mortality and recurrent MI, respectively. Therefore, the results should be interpreted with caution. Secondly, the definition of elevated TMAO levels varies among different studies. For example, Zhao et al. (39) considered 2.86 μmol as cut-off value of TMAO level. Zhou et al. (31) reported that the cut-off value of TMAO were classified into “high” (≥4.5 μmol) or “low” (<4.5 μmol). Therefore, our results could be unstable due to the different definition of elevated TMAO level. Thirdly, environmental factors (e.g., dietary intake) could influence the gut microbiota (51, 52) and might, this in turn affects the levels of TMAO and its precursors in the blood (46). However, only a detailed assessment of dietary intake was provided in some included studies. Therefore, further research is needed to investigate the potential role of nutritional factors in the metabolism of gut microbiota and prognosis in MI patients. Finally, most participants in all the included studies were from Asia, USA, and Europe. Therefore, our findings may not apply to other ethnic populations, such as Australians and Africans. The relationship between TMAO and MI prognosis should also be examined among Australians and Africans.

## Conclusion

TMAO could be an effective biomarker for adverse cardiovascular events. Our research results show a significant positive correlation between TMAO levels and MACE, all-cause mortality, and recurrent MI after MI. Further research needs to investigate whether TMAO's precursors might influence the prognosis in patients with MI.

## Data Availability

The original contributions presented in the study are included in the article/Supplementary Material, further inquiries can be directed to the corresponding author.

## Supplementary material

Supplementary Figure S1 Sensitivity analysis of trimethylamine-N-oxide levels in major adverse cardiovascular events after myocardial infarction.

Supplementary Figure S2 Sensitivity analysis of trimethylamine-N-oxide levels in all-cause mortality after myocardial infarction.

Supplementary Figure S3 Sensitivity analysis of trimethylamine-N-oxide levels in recurrent myocardial infarction after myocardial infarction.

Supplementary Figure S4 Subgroup analyses of the association between trimethylamine-N-oxide levels on major adverse cardiovascular according to study design (A) and geographical location of populations (B).

Supplementary Figure S5 Funnel plot in major adverse cardiovascular events (A), all-cause mortality (B), and recurrent myocardial infarction (C).
