# Supplementary figures and images for: Association between trimethylamine N-oxide and prognosis of patients with myocardial infarction: a meta-analysis
Source: Front Cardiovasc Med. 2024 Dec 10;11:1334730. doi: 10.3389/fcvm.2024.1334730 (PMC11666687; doi:10.3389/fcvm.2024.1334730)

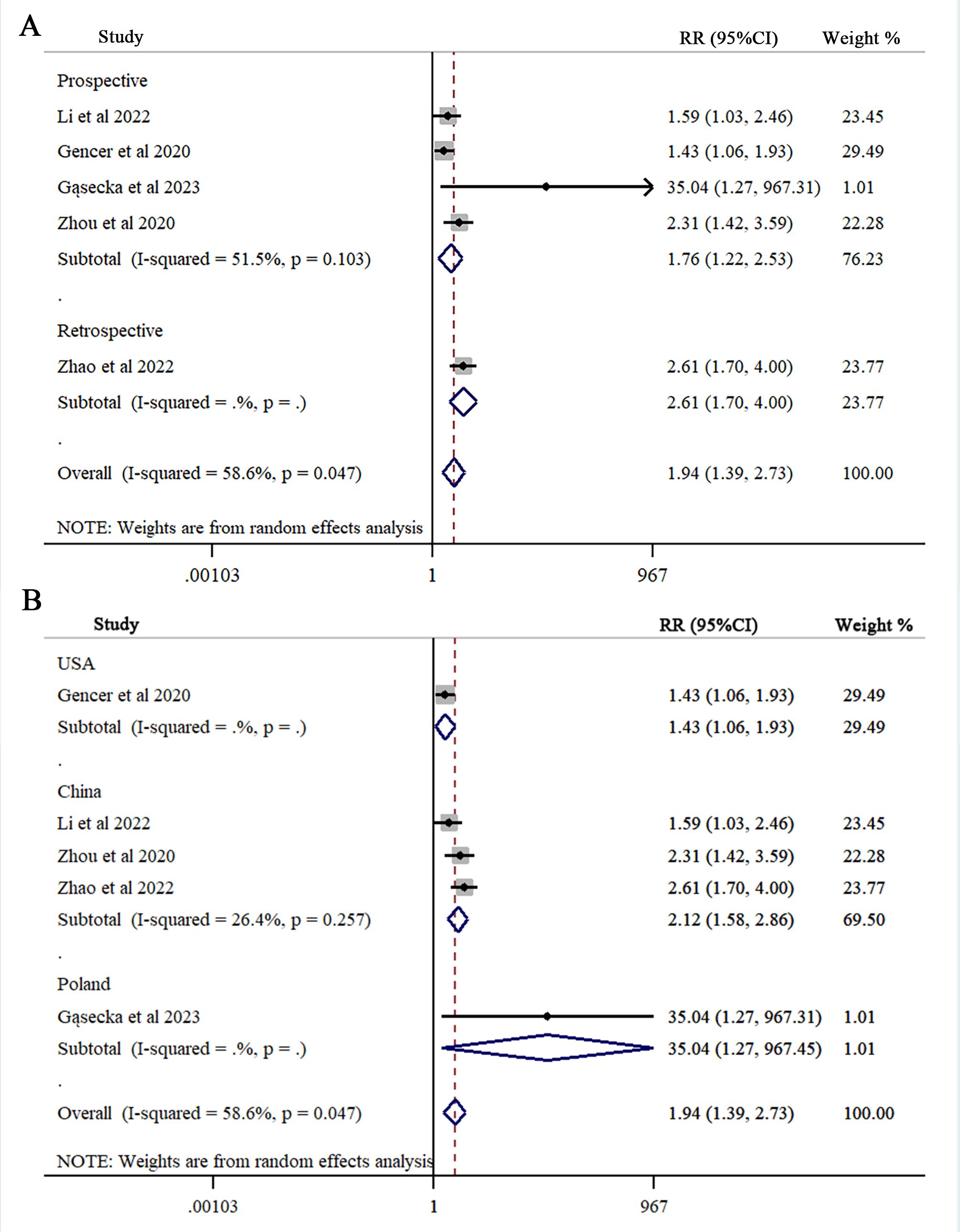

Supplement: Supplementary Figure S1 — Sensitivity analysis of trimethylamine-N-oxide levels in major adverse cardiovascular events after myocardial infarction. [file Image1.tif]

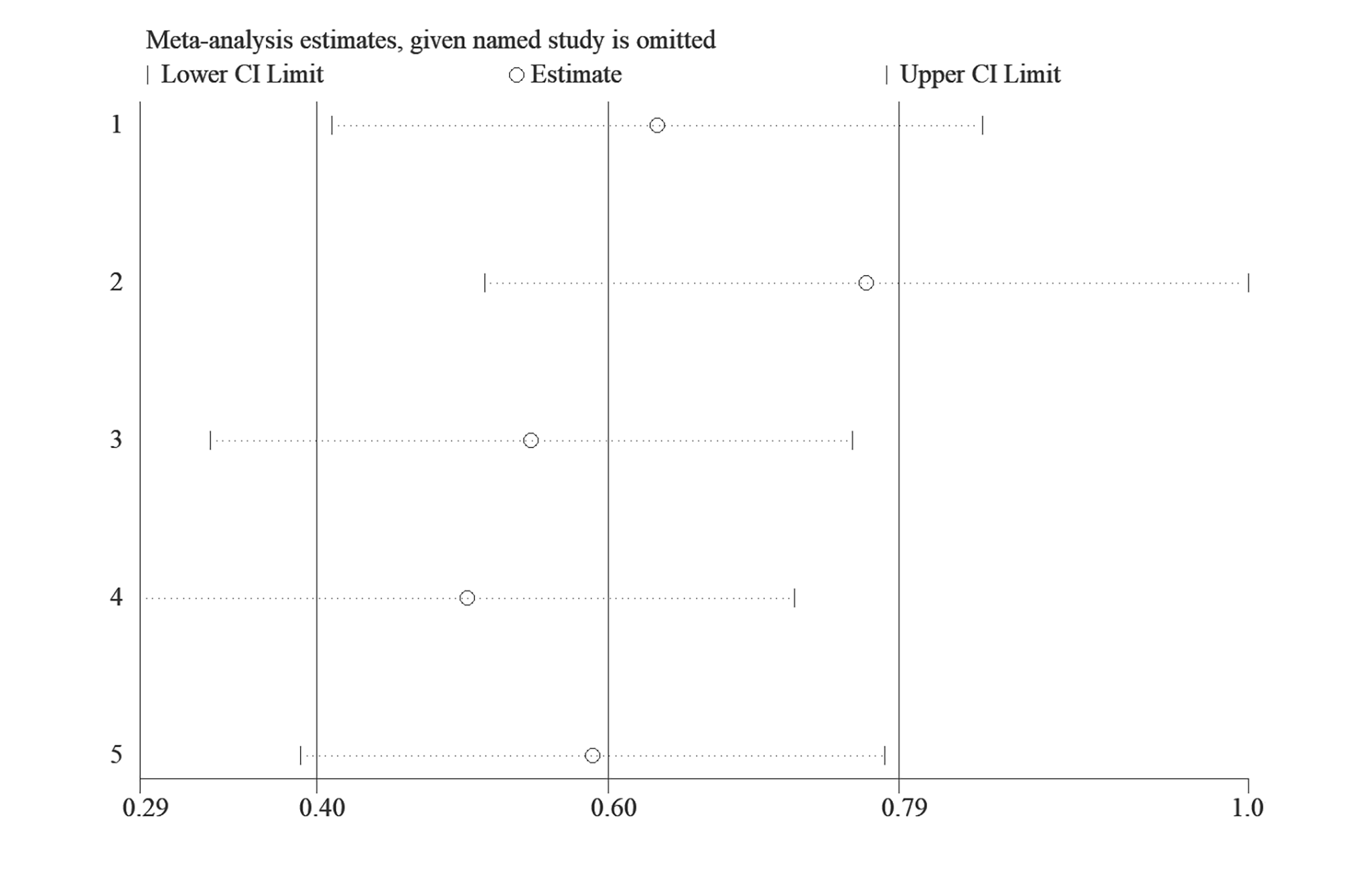

Supplement: Supplementary Figure S2 — Sensitivity analysis of trimethylamine-N-oxide levels in all-cause mortality after myocardial infarction. [file Image2.tif]

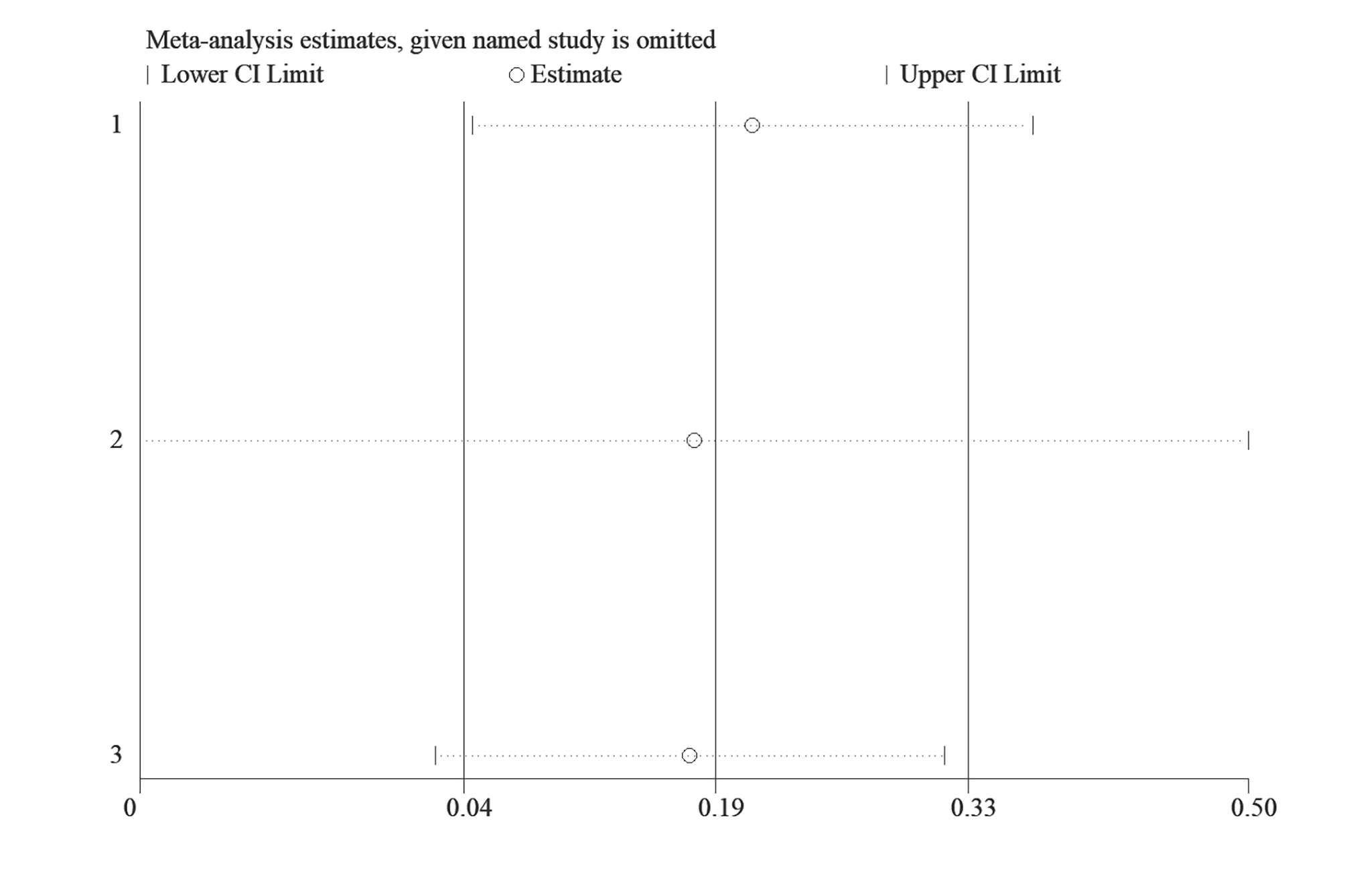

Supplement: Supplementary Figure S3 — Sensitivity analysis of trimethylamine-N-oxide levels in recurrent myocardial infarction after myocardial infarction. [file Image3.tif]

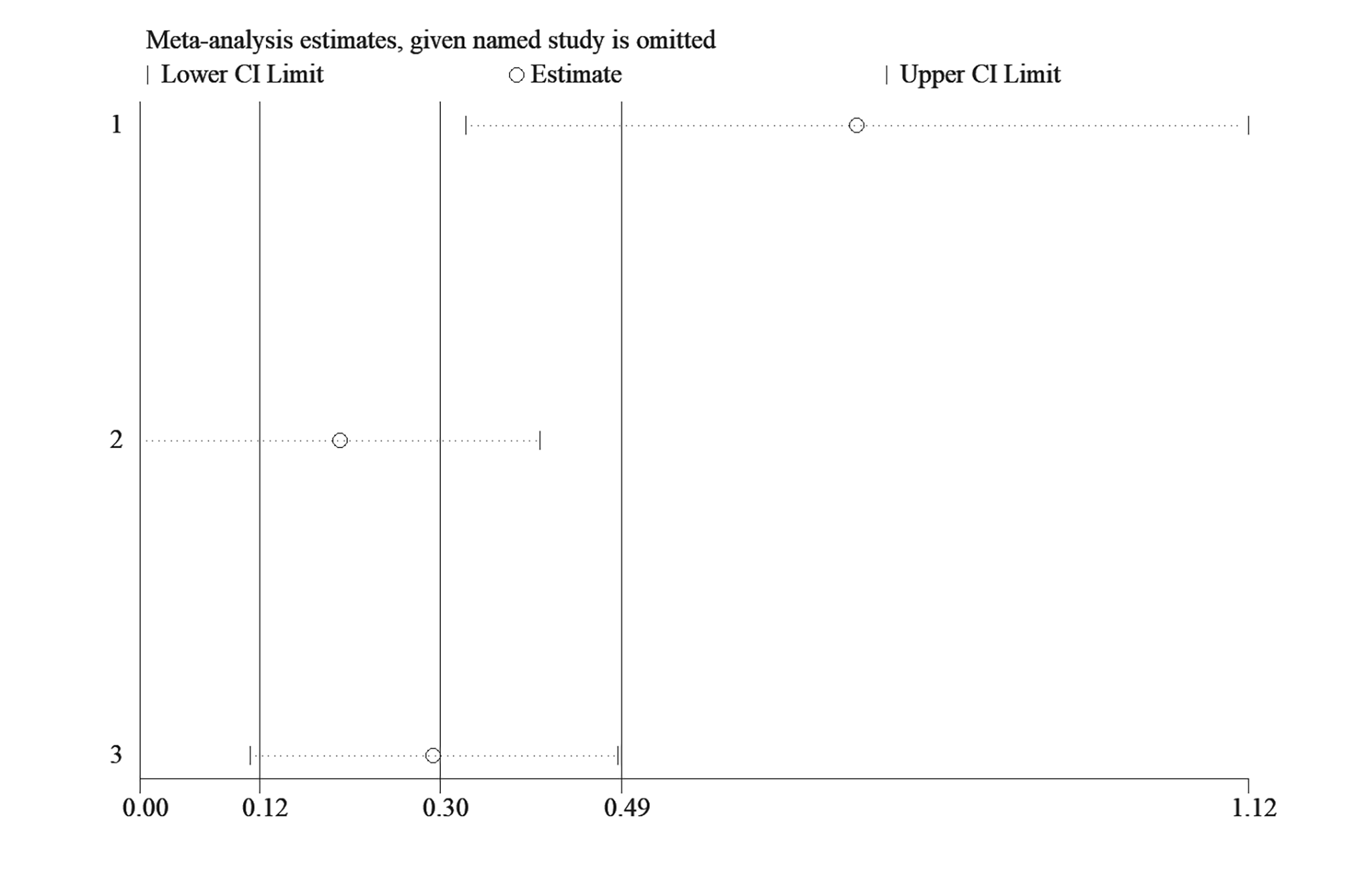

Supplement: Supplementary Figure S4 — Subgroup analyses of the association between trimethylamine-N-oxide levels on major adverse cardiovascular according to study design (A) and geographical location of populations (B). [file Image4.tif]

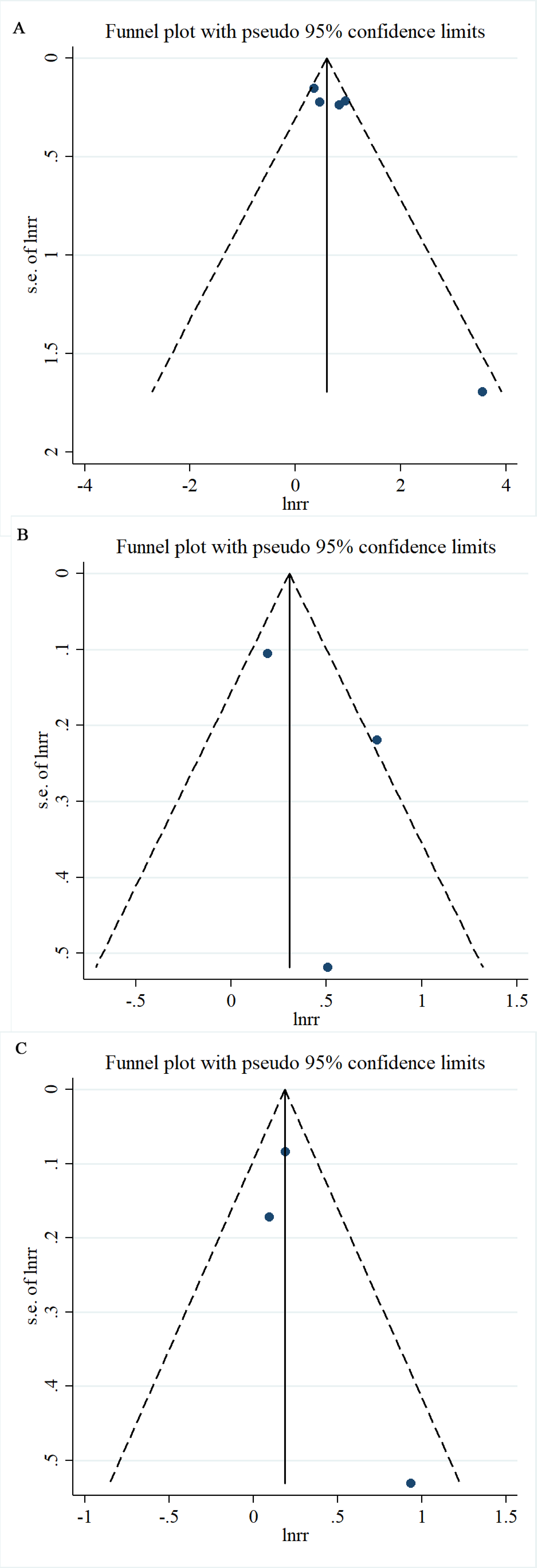

Supplement: Supplementary Figure S5 — Funnel plot in major adverse cardiovascular events (A), all-cause mortality (B), and recurrent myocardial infarction (C). [file Image5.tif]
